# Reintubation in the ICU: A Prospective Observational Study of Its Impact on Recovery Outcomes and Mortality

**DOI:** 10.7759/cureus.115813

**Published:** 2026-09-05

**Authors:** Renu Wakhloo, Zahida Aziz, Preeti Sharma, Siva Shree, Ranika Manhas, Sunny Babber

**Affiliations:** 1 Anesthesiology and Critical Care, Government Medical College and Hospital, Jammu, IND; 2 Community Medicine, Acharya Shri Chander College of Medical Sciences and Hospital, Jammu, IND

**Keywords:** extubation failure, intensive care unit, mechanical ventilation, mortality, rapid shallow breathing index, reintubation

## Abstract

Background

Extubation is an important step in the recovery of critically ill patients, but some patients deteriorate after removal of the endotracheal tube and require reintubation. Extubation failure can prolong intensive care and is associated with poor outcomes. This study examined the factors associated with reintubation and the clinical outcomes of patients who failed planned extubation.

Methods

We prospectively followed adult patients undergoing planned extubation in the adult ICU of a tertiary care hospital between June 2025 and June 2026. Patients were observed for 72 hours after extubation, and those requiring reintubation during this period were considered to have experienced extubation failure. Demographic, clinical, physiological, and laboratory characteristics were compared between patients who remained successfully extubated and those who required reintubation. Factors associated with reintubation and in-hospital mortality were examined using multivariable logistic regression.

Results

A total of 180 patients were included, of whom 27 (15.0%) required reintubation within 72 hours. The patients who required reintubation were older and had higher Acute Physiology and Chronic Health Evaluation II (APACHE II) and Rapid Shallow Breathing Index (RSBI) scores, while their Glasgow Coma Scale (GCS) scores and serum albumin levels were lower. Positive fluid balance, emergency admission, congestive heart failure, chronic lung disease, and acute kidney injury requiring renal replacement therapy were also more frequent in this group. Reintubation was associated with a longer duration of mechanical ventilation, longer ICU and hospital stays, and higher rates of ventilator-associated pneumonia and tracheostomy. ICU and in-hospital mortality were also considerably higher among patients who required reintubation. After adjustment for other factors, APACHE II score >20, GCS <12, RSBI >105, serum albumin <3.5 g/dL, positive fluid balance >1 L, and mechanical ventilation >7 days were associated with reintubation. Reintubation was strongly associated with in-hospital mortality (adjusted OR, 5.84; 95% CI, 2.31-14.78; P < 0.001).

Conclusions

In this cohort, patients who required reintubation after planned extubation had a substantially more difficult clinical course and a higher risk of death. Greater illness severity, poor respiratory reserve, low serum albumin, positive fluid balance, and prolonged mechanical ventilation were associated with extubation failure. Identifying these features before extubation and maintaining close observation after extubation may help clinicians recognize patients at higher risk of deterioration.

## Introduction

Discontinuation of invasive mechanical ventilation is a major milestone in the recovery of critically ill patients admitted to the ICU. Successful extubation reflects adequate respiratory function, neurological recovery, airway protection, and hemodynamic stability, whereas extubation failure requiring reintubation is associated with increased morbidity, prolonged mechanical ventilation, ventilator-associated pneumonia (VAP), longer ICU and hospital stays, higher healthcare costs, and increased mortality [1-4]. Despite advances in weaning strategies and spontaneous breathing trials, extubation failure continues to occur in approximately 10-20% of critically ill adults [5,6]. Delayed recognition of extubation failure and delayed reintubation further worsen clinical outcomes, including prolonged respiratory support, increased complications, and mortality [7].

Several clinical and physiological variables, including the Rapid Shallow Breathing Index (RSBI), Glasgow Coma Scale (GCS), Acute Physiology and Chronic Health Evaluation II (APACHE II) score, serum albumin concentration, cumulative fluid balance, cough strength, airway secretion burden, and underlying cardiopulmonary disease, have been evaluated as predictors of extubation success. Extubation readiness is generally assessed following a successful spontaneous breathing trial, with adequate oxygenation (SpO₂ close to baseline and PaCO₂ within or close to baseline), adequate neurologic status, airway protection, and hemodynamic stability. However, no single predictor has demonstrated adequate accuracy [8-13]. Previous studies have also shown that reintubation independently increases the risk of VAP, tracheostomy, prolonged ICU stay, and mortality [14].

Data from Indian ICUs remain limited. Therefore, this study evaluated predictors, causes, and outcomes of reintubation following planned extubation and identified factors independently associated with in-hospital mortality.

## Materials and methods

This was a prospective observational study carried out in the Department of Anesthesia and Critical Care, Government Medical College and Hospital, Jammu, from June 2025 to June 2026 in the adult ICU. Adult patients who had been on invasive mechanical ventilation for >48 hours and were planned for extubation were included. A 72-hour post-extubation follow-up was conducted for all patients. Extubation failure was considered to be reintubation during this period. Patients who self-extubated or were accidentally extubated, those with a preexisting tracheostomy, and those who were reintubated for reasons other than extubation failure were excluded. Age at extubation, sex, BMI, APACHE II score [15], GCS [16], RSBI [17], serum albumin level, fluid balance, type of hospitalization to the ICU, and pertinent comorbidities at the time of extubation were recorded. The clinical course was also followed, including duration of mechanical ventilation, length of ICU and hospital stay, VAP, tracheostomy, and mortality. The time from extubation to reintubation and the clinical indication for reintubation were documented for patients who required reintubation.

The minimum sample size was determined to be 136 patients, based on a previously reported reintubation rate of 15%. To increase the precision of the estimates, the final sample size was increased to 180 patients. Continuous variables were expressed as mean ± SD or median and IQR, as appropriate. The independent-samples t-test was used to compare normally distributed continuous variables between the two groups, while the Mann-Whitney U test was used for nonnormally distributed continuous variables. Categorical variables were expressed as numbers and percentages and compared using Pearson’s chi-square test or Fisher’s exact test, as appropriate. Factors associated with reintubation and in-hospital mortality were identified using multivariable logistic regression analysis. ORs with 95% CIs were reported. Two-sided P-values <0.05 were considered statistically significant. The study was expedited-reviewed by the Institutional Ethics Committee of Government Medical College, Jammu (IEC/GMCJ/2025/2699).

Figure 1 shows the participant flow diagram of the study.

**Figure 1:**
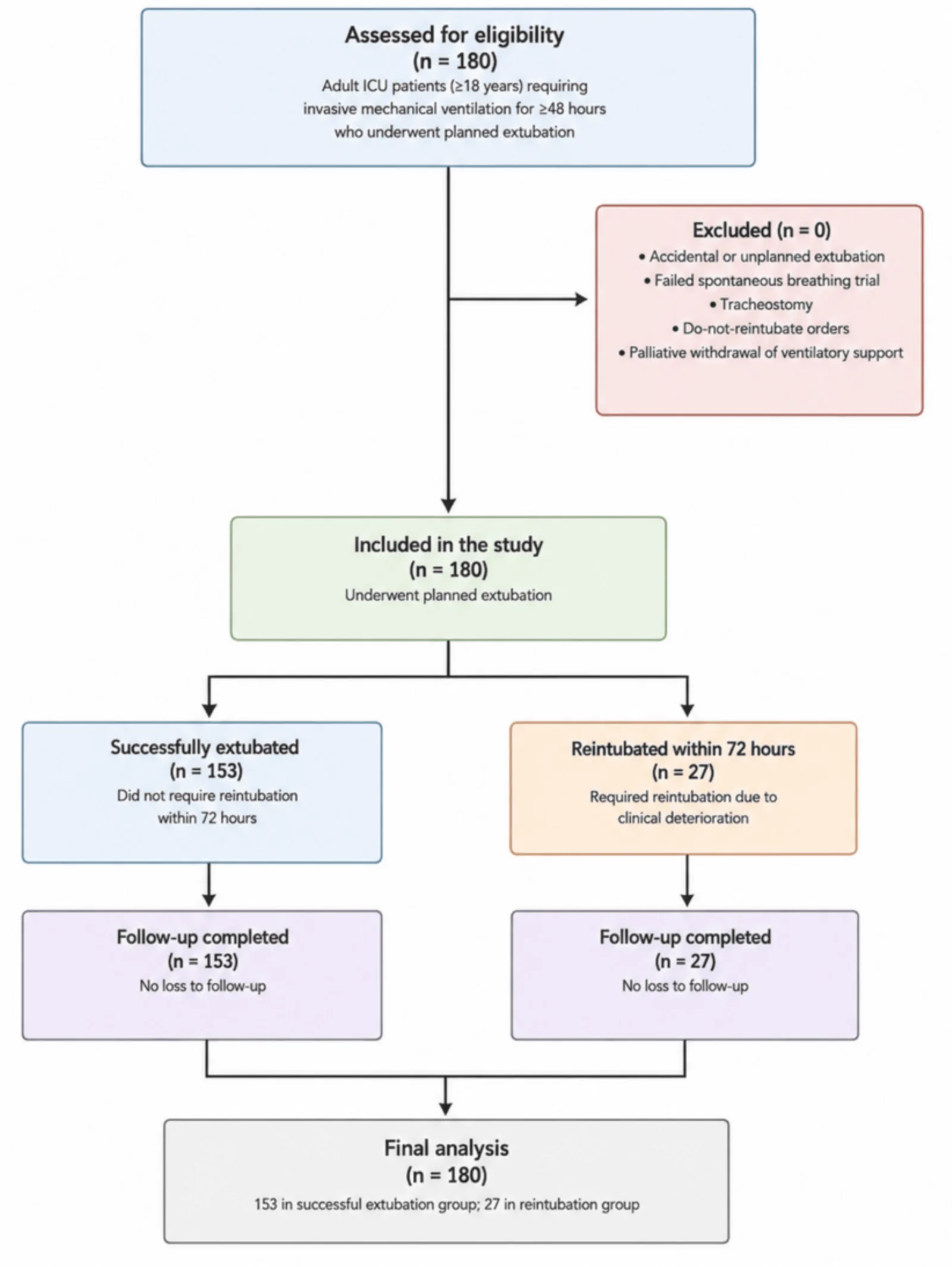
Participant flow diagram showing patient selection, planned extubation, reintubation within 72 hours, follow-up, and final analysis. This figure was created using Microsoft Excel (Microsoft Corporation, Redmond, WA, USA).

## Results

Table 1 shows that patients requiring reintubation were significantly older (P = 0.001) and had higher APACHE II scores and RSBI, while GCS and serum albumin levels were significantly lower (all P < 0.001). Positive fluid balance >1 L (P = 0.002), emergency admission (P = 0.014), congestive heart failure (P = 0.016), chronic lung disease (P = 0.018), and acute kidney injury (AKI) requiring renal replacement therapy (P = 0.024) were significantly more frequent in the reintubation group. No significant differences were observed in sex (P = 0.742), BMI (P = 0.443), or diabetes mellitus (P = 0.274).

**Table 1:** Comparison of demographic, clinical, and laboratory characteristics between successfully extubated and reintubated patients. Values are mean ± SD or n (%). Continuous variables were compared using t-tests, and categorical variables were compared using Pearson’s χ² test or Fisher’s exact test, as appropriate. Test statistics are reported as χ² (df) for Pearson’s χ² tests; exact P-values are reported for Fisher’s exact tests. AKI, acute kidney injury; APACHE II, Acute Physiology and Chronic Health Evaluation II; GCS, Glasgow Coma Scale; RSBI, Rapid Shallow Breathing Index

Variable	Successfully extubated (n = 153)	Reintubated (n = 27)	Total (n = 180)	Statistical test, t/χ² (df)	P-value
Age (years), mean ± SD	56.2 ± 11.4	63.7 ± 10.2	57.3 ± 11.4	t = -3.26 (178)	0.001
Male sex, n (%)	97 (63.4)	18 (66.7)	115 (63.9)	χ² = 0.11 (1)	0.742
BMI (kg/m²), mean ± SD	25.7 ± 3.8	25.1 ± 3.6	25.6 ± 3.8	t = 0.77 (178)	0.443
APACHE II score, mean ± SD	15.8 ± 4.8	22.7 ± 5.1	16.8 ± 5.3	t = -6.82 (178)	<0.001
GCS, mean ± SD	13.8 ± 1.5	11.6 ± 1.7	13.5 ± 1.7	t = 6.89 (178)	<0.001
RSBI, mean ± SD	75.1 ± 20.8	109.8 ± 22.7	80.3 ± 24.4	t = -7.82 (178)	<0.001
Serum albumin (g/dL), mean ± SD	3.67 ± 0.42	3.04 ± 0.38	3.58 ± 0.46	t = 7.52 (178)	<0.001
Positive fluid balance >1 L, n (%)	31 (20.3)	13 (48.1)	44 (24.4)	χ² = 9.55 (1)	0.002
Emergency admission, n (%)	86 (56.2)	22 (81.5)	108 (60.0)	χ² = 6.04 (1)	0.014
Diabetes mellitus, n (%)	41 (26.8)	10 (37.0)	51 (28.3)	χ² = 1.20 (1)	0.274
Congestive heart failure, n (%)	11 (7.2)	6 (22.2)	17 (9.4)	χ² = 5.80 (1)	0.016
Chronic lung disease, n (%)	18 (11.8)	8 (29.6)	26 (14.4)	χ² = 5.60 (1)	0.018
AKI requiring renal replacement therapy, n (%)	9 (5.9)	5 (18.5)	14 (7.8)	χ² = 5.10 (1)	0.024

The distribution of primary ICU diagnoses did not differ significantly between the two groups (P = 0.378). Patients requiring reintubation had significantly longer durations of mechanical ventilation, ICU stays, and hospital stays (all P < 0.001). VAP, tracheostomy, ICU mortality, and in-hospital mortality were significantly more frequent among patients requiring reintubation (all P < 0.001), as shown in Table 2.

**Table 2:** Comparison of primary ICU diagnosis and clinical outcomes between successfully extubated and reintubated patients. Values are mean ± SD or n (%). Continuous variables were compared using t-tests, and categorical variables were compared using Pearson’s χ² test or Fisher’s exact test, as appropriate. Test statistics are reported as χ² (df) for Pearson’s χ² tests; exact P-values are reported for Fisher’s exact tests. VAP, ventilator-associated pneumonia

Variable	Successfully extubated (n = 153)	Reintubated (n = 27)	Statistical test, t/χ² (df)	P-value
Primary diagnosis at ICU admission, n (%)				
Respiratory disease	42 (27.5)	11 (40.7)	χ² = 5.32 (5)	0.378
Sepsis	29 (19.0)	5 (18.5)		
Neurological disease	23 (15.0)	6 (22.2)		
Postoperative care	34 (22.2)	2 (7.4)		
Trauma	13 (8.5)	1 (3.7)		
Cardiovascular disease	12 (7.8)	2 (7.4)		
Duration of mechanical ventilation before extubation (days), mean ± SD	5.3 ± 2.4	8.4 ± 3.1	t = -5.91 (178)	<0.001
ICU length of stay (days), mean ± SD	10.2 ± 4.7	21.3 ± 8.2	t = -9.93 (178)	<0.001
Hospital length of stay (days), mean ± SD	20.8 ± 9.1	32.8 ± 12.4	t = -5.96 (178)	<0.001
VAP, n (%)	16 (10.5)	10 (37.0)	χ² = 13.12 (1)	<0.001
Tracheostomy, n (%)	6 (3.9)	7 (25.9)	χ² = 16.58 (1)	<0.001
ICU mortality, n (%)	8 (5.2)	9 (33.3)	χ² = 21.20 (1)	<0.001
In-hospital mortality, n (%)	12 (7.8)	11 (40.7)	χ² = 22.29 (1)	<0.001

Among the 27 patients who required reintubation, 14 (51.9%) were reintubated within ≤12 hours, eight (29.6%) within >12-24 hours, and five (18.5%) within >24-72 hours. Thus, most reintubation events occurred during the first 12 hours following extubation, as shown in Figure 2.

**Figure 2:**
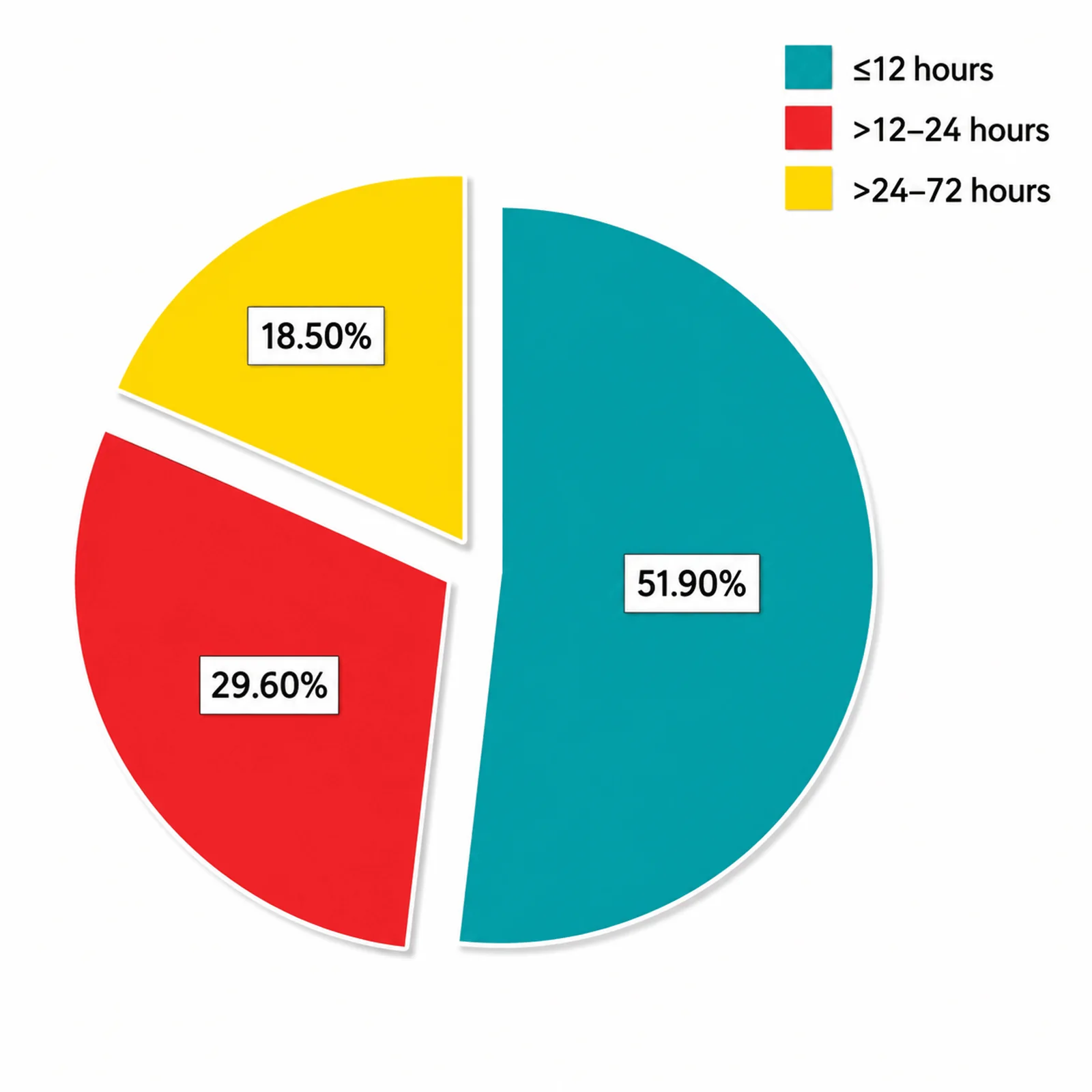
Timing of reintubation within 72 hours following planned extubation. This figure was created using IBM SPSS Statistics for Windows, version 21.0 (released 2012; IBM Corp., Armonk, NY, USA).

Figure 3 shows that among the 27 patients who required reintubation, respiratory muscle fatigue was the most common cause of extubation failure, accounting for six (22.2%) cases, followed by hypoxemic respiratory failure in five (18.5%) and upper airway obstruction in five (18.5%). Neurological deterioration accounted for four (14.8%) cases, while excessive airway secretions accounted for three (11.1%). Hypercapnic respiratory failure occurred in two (7.4%) patients, and hemodynamic instability and metabolic disturbance accounted for one (3.7%) case each.

**Figure 3:**
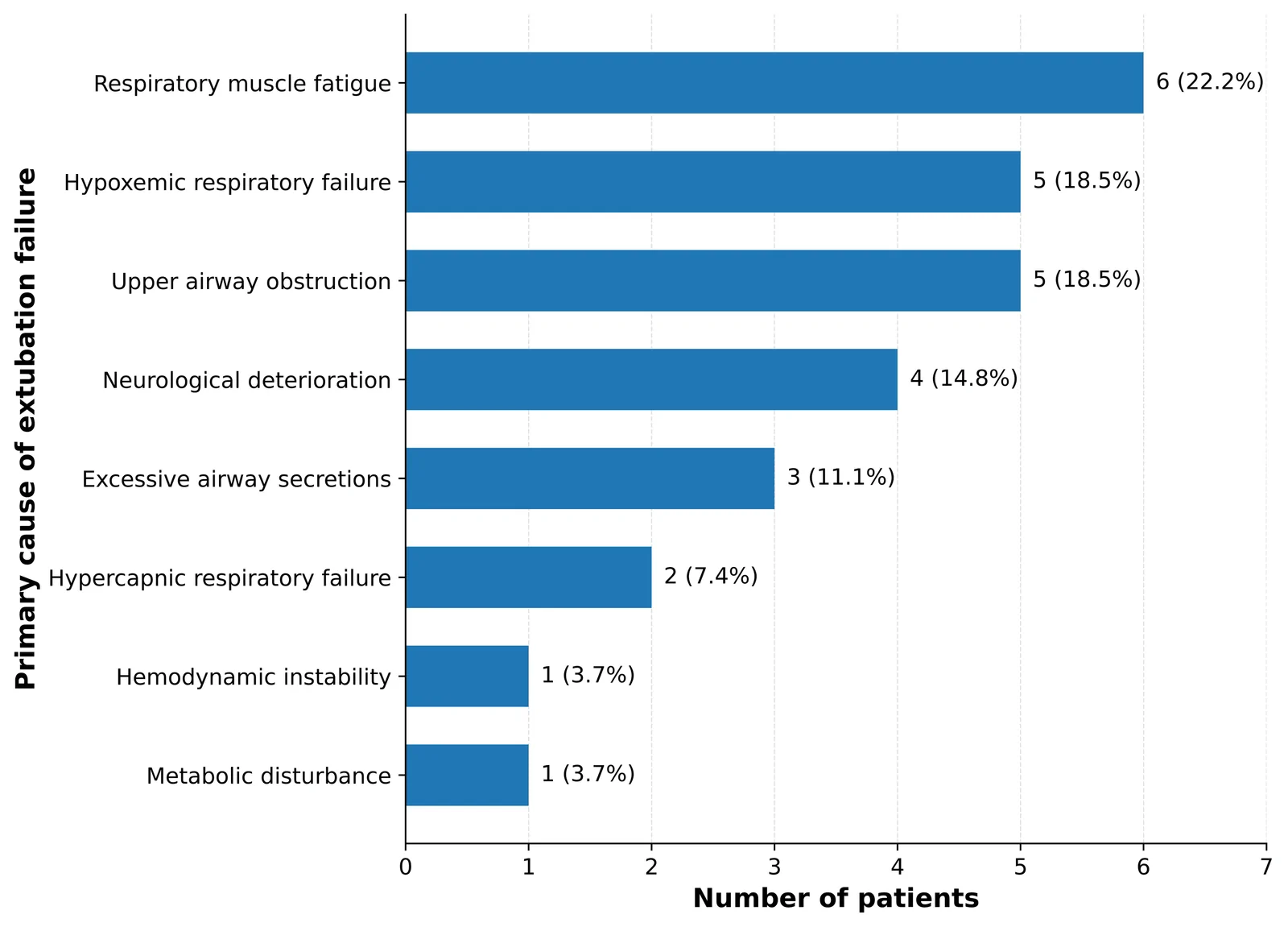
Primary causes of extubation failure among patients requiring reintubation within 72 hours. This figure was created using IBM SPSS Statistics for Windows, version 21.0 (released 2012; IBM Corp., Armonk, NY, USA).

Table 3 presents the multivariable logistic regression analyses for reintubation and in-hospital mortality. For reintubation, APACHE II score >20, GCS <12, RSBI >105, serum albumin <3.5 g/dL, positive fluid balance >1 L, and mechanical ventilation >7 days were independently associated with increased odds of reintubation. Among these factors, RSBI >105 showed the strongest association, followed by APACHE II score >20. For in-hospital mortality, reintubation was independently associated with substantially higher odds of mortality. APACHE II score >20, VAP, AKI requiring renal replacement therapy, and mechanical ventilation >7 days were also independently associated with increased odds of in-hospital mortality. Age >65 years, serum albumin <3.5 g/dL, GCS <12, diabetes mellitus, and congestive heart failure were not independently associated with mortality after multivariable adjustment.

**Table 3:** Multivariable logistic regression analysis of factors associated with reintubation and in-hospital mortality. Wald χ² values shown are calculated from (β/SE)² based on the reported regression coefficients and standard errors. Statistical significance was defined as P < 0.05. AKI, acute kidney injury; APACHE II, Acute Physiology and Chronic Health Evaluation II; β, regression coefficient; GCS, Glasgow Coma Scale; RSBI, Rapid Shallow Breathing Index; VAP, ventilator-associated pneumonia

Outcome	Variable	β	SE	Wald χ² (df)	Adjusted OR (95% CI)	P-value
Reintubation	Age >65 years	0.574	0.352	2.66 (1)	1.78 (0.89-3.57)	0.103
	APACHE II score >20	1.509	0.446	11.45 (1)	4.52 (1.88-10.89)	<0.001
	GCS <12	1.227	0.456	7.24 (1)	3.41 (1.39-8.37)	0.007
	RSBI >105	1.728	0.463	13.93 (1)	5.63 (2.27-13.94)	<0.001
	Serum albumin <3.5 g/dL	1.034	0.469	4.86 (1)	2.81 (1.12-7.06)	0.028
	Positive fluid balance >1 L	0.967	0.454	4.54 (1)	2.63 (1.08-6.42)	0.034
	Mechanical ventilation >7 days	1.125	0.467	5.80 (1)	3.08 (1.24-7.67)	0.016
	Emergency admission	0.641	0.417	2.36 (1)	1.90 (0.84-4.28)	0.124
	Congestive heart failure	0.698	0.491	2.02 (1)	2.01 (0.77-5.25)	0.155
	Chronic lung disease	0.793	0.476	2.78 (1)	2.21 (0.87-5.61)	0.096
In-hospital mortality	Reintubation	1.765	0.474	13.87 (1)	5.84 (2.31-14.78)	<0.001
	Age >65 years	0.761	0.404	3.55 (1)	2.14 (0.98-4.66)	0.056
	APACHE II score >20	1.23	0.435	8.00 (1)	3.42 (1.46-8.04)	0.005
	VAP	1.008	0.453	4.95 (1)	2.74 (1.12-6.69)	0.027
	AKI requiring renal replacement therapy	1.185	0.514	5.32 (1)	3.27 (1.19-8.96)	0.021
	Mechanical ventilation >7 days	0.853	0.428	3.97 (1)	2.35 (1.02-5.42)	0.046
	Serum albumin <3.5 g/dL	0.842	0.437	3.71 (1)	2.32 (0.99-5.44)	0.054
	GCS <12	0.594	0.463	1.65 (1)	1.81 (0.73-4.48)	0.199
	Diabetes mellitus	0.382	0.417	0.84 (1)	1.46 (0.64-3.35)	0.359
	Congestive heart failure	0.721	0.471	2.34 (1)	2.06 (0.82-5.17)	0.125

## Discussion

In this prospective observational study, 15.0% (n = 27) of the 180 patients needed reintubation within 72 hours of planned extubation. Patients requiring reintubation were significantly more ill at extubation, with increased APACHE II and RSBI and decreased GCS and serum albumin levels, as well as increased positive fluid balance. Mechanical ventilation duration, ICU and hospital stays, development of VAP, and tracheostomy were also associated with reintubation, as was a significantly higher mortality rate in the ICU and hospital. The results indicate that extubation failure is typically multifactorial and is a manifestation of limited physiological reserve, not a single abnormality.

Current guidelines recommend considering extubation readiness beyond the spontaneous breathing trial [18]. APACHE II score >20, GCS <12, and RSBI >105 were independently associated with reintubation in our study. The median RSBI in the reintubation group was 109, while that in the successfully extubated group was 72, and RSBI was the most predictive factor for reintubation (adjusted OR, 5.63; 95% CI, 2.27-13.94; P < 0.001). These results agree with previous reports that the outcome of extubation is related to a combination of clinical and physiological factors [19,20].

More than half (51.9%) of the reintubations were performed within the first 12 hours after extubation and 81.5% within the first 24 hours, making the first post-extubation day the most critical time for reintubation. The most frequent cause of reintubation was respiratory muscle fatigue (22.2%), followed by hypoxemic respiratory failure and upper airway obstruction (18.5% for both causes). This underlines the importance of careful monitoring, particularly during the early post-extubation phase. Results from high-risk patients also indicate a possible role for adequate post-extubation respiratory assistance [21]. Previous studies have emphasized the importance of the timing of reintubation [22].

Although previous studies reported sex differences in extubation outcomes [23], sex was not significantly associated with reintubation in our cohort. Additional factors independently associated with reintubation were positive fluid balance >1 L and serum albumin <3.5 g/dL. These factors may exacerbate pulmonary congestion and increase the work of breathing, while low albumin may indicate decreased physiological reserve, and the variety of causes seen in our cohort mirrors the multifactorial nature of extubation failure [24].

Another significant finding was prolonged mechanical ventilation. Compared with successfully extubated patients, patients requiring reintubation had a median ventilation duration of eight days. Mechanical ventilation >7 days was an independent predictor of reintubation and in-hospital mortality. This may be due to respiratory muscle and diaphragmatic weakness and more severe disease associated with prolonged ventilation [25]. It has previously been reported that fluid balance is associated with weaning outcomes [26].

Importantly, reintubation was associated with significantly higher mortality (adjusted OR, 5.84; 95% CI, 2.31-14.78; P < 0.001). Therefore, these easily available clinical data may be used to identify patients who need more intensive monitoring following extubation [27]. A strength of this study is its prospective design and systematic evaluation of clinical and physiological parameters.

Limitations

The results may not be generalizable, however, because of the single-center design, relatively small number of reintubation events, and differences in extubation practices across ICUs and populations. Because this is an observational study, the associations found should not be considered causal. Further multicenter research is necessary to confirm these results and to determine whether a focused approach may decrease extubation failure and improve outcomes.

## Conclusions

Patients requiring reintubation after planned extubation had longer durations of mechanical ventilation, longer ICU and hospital stays, higher incidences of VAP and tracheostomy, and higher ICU and in-hospital mortality. Higher APACHE II scores, increased RSBI, lower GCS, hypoalbuminemia, positive cumulative fluid balance, and prolonged mechanical ventilation were independently associated with an increased risk of reintubation. Reintubation was also independently associated with higher in-hospital mortality. Early identification of high-risk patients, optimization of modifiable risk factors, and vigilant post-extubation monitoring may improve extubation outcomes and clinical outcomes.
